# The role of social support and the built environment on diabetes management among structurally exposed populations in three regions in Ghana

**DOI:** 10.1186/s12889-023-17376-y

**Published:** 2023-12-13

**Authors:** Joseph Kangmennaang, Alhassan Siiba, Ebenezer Dassah, Moses Kansanga

**Affiliations:** 1 School of Kinesiology and Health Studies, Queen’s University, Building 28 Division Street, Kingston, ON K7L 3N6 Canada; 2 Department of Global and International Health, Kwame Nkrumah University of Science and Technology, Ghana Post GPS AK-385-19, Kumasi, Ghana; 3 Department of Geography, The George Washington University, Samson Hall, Second Floor 2036 H St. NW, Washington, D.C, 20052 USA

**Keywords:** Diabetes management, Social support, Built environment, Epidemiological transition

## Abstract

Sub-Saharan Africa is undergoing an epidemiological transition driven by rapid, unprecedented demographic, socio-cultural, and economic transitions. These transitions are driving increases in the risk and prevalence of diabetes and other non-communicable diseases (NCDs). As NCDs rise, several attempts have been made to understand the individual level factors that increase NCDs risks, knowledge, and attitudes around specific NCDs as well as how people live and manage NCDs. While these studies are important, and enhance knowledge on chronic diseases, little attention has been given to the role of social and cultural environment in managing chronic NCDs in underserved settings. Using purposive sampling among persons living with Diabetes Mellitus (PLWD) and participating in diabetes programs from regional and municipal hospitals in the three underserved regions in Ghana (*n *= 522), we assessed diabetes management and supportive care needs of PLWDs using linear latent and mixed models (gllamm) with binomial and a logit(log) link function. The result indicates that PLWDs with strong perceived social support (OR = 2.27, *p* ≤ 0.05) were more likely to report good diabetes management compared to PLWDs with weak perceived social support. The built environment, living with other health conditions, household wealth, ethnicity and age were associated with diabetes management. Overall, the study contributes to wider discussions on the role changing built and socio-cultural environments in the rise of diet-related diseases and their management as many Low- and Middle-Income Countries (LMICs) experience rapid epidemiological and nutrition transitions.

## Introduction

Diabetes is a leading cause of death in the world. As at the year 2019, about 463 million of the global population had diabetes with projections further indicating that the number could rise by 51% to reach 700 million people by 2045 [1]. Sub-Saharan Africa (SSA) is undergoing an epidemiological transition driven by rapid, unprecedented demographic, socio-cultural, and economic transformations which are driving increases in the risk and prevalence of diabetes and other non-communicable diseases (NCDs). About 24 million adults living with Diabetes Mellitus (PLWD) globally are found in sub-Saharan Africa (SSA), and this figure is predicted to rise to 55 million by 2045, an increase of 134%, with young people accounting for a disproportionate percentage of PLWD [1]. Uncontrolled diabetes is associated with increased risk for severe health complications, including but not limited to, heart disease, chronic kidney disease, nerve damage, and other related problems with oral health, vision, hearing, and mental health [2, 3]. Data from surveys conducted in 28 low- and middle-income countries (LMICs) showed that of all patients with Type 2 Diabetes, only 38% are on treatment, and 23% achieve glycemic control [4]. Early diagnosis and conscious control of unhealthy lifestyle and habits are some recommended measures for managing the negative impacts of diabetes [5, 6]. However, managing the inherent negative impacts of diabetes is dependent upon the availability of resources including access to screening and information, social support, and supportive socio-political environments [7–9]. The intersection of personal responsibility for diabetes management and low incomes in SSA, coupled with the new challenges of COVID-19 predisposes PLWD to inadequate care and poor glycemic controls due to the disruption of the healthcare system and the economy. In SSA, adequate supply of diabetes medication and promoting lifestyle changes are crucial to minimize routine hospital visits, reduce diabetes related depression and promote the general wellbeing of PLWD. Thus, to adequately manage the disease, PLWD do not only need timely care, diabetes self-management education and self-management support but social support as well. Social support may help improve adherence to anti-hyperglycemic medications leading to improved health outcomes.

Diabetes self-management forms a significant part of diabetes management, and there is evidence that adults with diabetes who perform self-management activities experience better health and wellbeing outcomes [10, 11]. Effective self-management of diabetes typically involves a complex regimen including healthy eating, weight control, medications, blood glucose monitoring, exercise, and stress management over long periods of time [12]. Diabetes self-management behaviors are influenced by cultural and lifestyle factors including food choices [12]. Impediments to diabetes self-management among PLWD in SSA include poor understanding of the relationship between diabetes and diet, poor psychological adjustment, denial that diabetes is serious, lack of confidence, coping skills, and competence [12, 13]. Negative attitudes, and emotional distresses such as depression and anxiety also contribute to poor diabetic control [14–16]. Apart from clinical factors, the ability to properly self-manage diabetes also depends on sociodemographic and socialcultural factors including family support. A systematic review found that lack of social support, lack of knowledge, and divergent cultural and spiritual values hinder patients from effectively managing their diabetes condition [14]. The changing sociocultural landscape in most LMICs, especially among rural and underserved populations, could also undermine social support leading to poor diabetes self-management.

The literature on diabetes in Ghana for instance, has focused on patients’ knowledge of diabetes, and very few studies have assessed patients’ support systems [17–19]. Further, evidence on the role that support systems play in improving dietary management, reducing diabetes complications as well as improving the general wellbeing of PLWD is limited [19]. To the best of our knowledge, apart from Botchway and colleagues’ study in urban Ghana [19], there is no other study focused on social support and diabetes management in the context of Ghana. As such, this study seeks to assess diabetes management and supportive care needs of PLWD in selected underserved communities in Ghana. The high burden of NCDs on the global front has been recognized and included in the United Nations' Sustainable Development Goals [20]. Specifically, Goal 3 of the UN SDGs is focused on health with target 3.4 aimed at reducing, by one-third, premature mortality from NCDs through prevention and treatment and promotion of mental health and wellbeing [20]. Despite the recognition of the challenges posed by NCDs, they remain underfunded and less prioritized and healthcare systems are not re-oriented to deal with NCDs in most LMICs [2, 4, 5]. In the resource poor setting, creating supportive communities and networks of support among PLWDs may contribute towards enhancing self-care and improving overall health and wellbeing [2, 20]. Thus, there is a potential value in designing interventions to enhance social support among PLWD to reduce depressive symptomatology, improve diet and medication adherence, and increase overall wellbeing.

### Theoretical framework

**Fig. 1:**
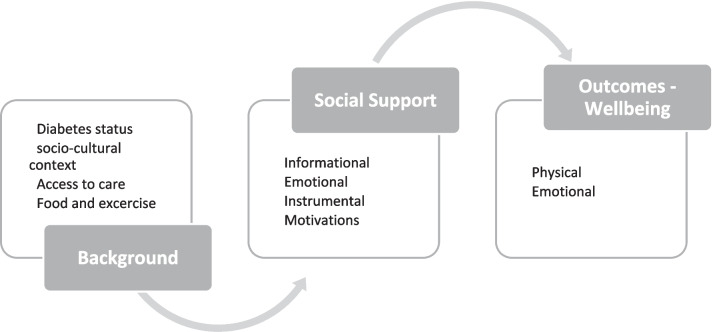
Overarching framework guiding the study

Social support helps patients with chronic conditions in significant ways to improve self-care, increase knowledge of disease symptoms, which may improve overall wellbeing. This study is informed by the Social Support Framework [21] as shown in Fig. 1. Social support is the perceived availability of functional support from family, friends, coworkers, and social networks including informational, emotional, and instrumental/tangible aid [21–23]. Informational social support involves helping PLWD to appreciate the stress or the cost associated with diabetes as well as the coping strategies they need to adopt to deal with their condition [24, 25]. Instrumental support involves the provision of physical assistance, to enable PLWD to put into action any informational support given them. Helping PLWDs with limited mobility to do regular exercise is an example of instrumental support [23]. When the impacts of informational and instrumental support are realized, PLWD are likely to feel some sense of emotional comfort that brings affection, reassuring them that they matter in their social networks irrespective of their health condition [25]. It should be noted, however, that these supports can be demonstrative or perceptive of their availability at the time of need [23, 26]. A person’s sense of belonging in a social group(s) has been tied to a myriad of health, social, psychological, and emotional benefits [7, 8, 26–30]. These studies generally note that PLWD who belong to or have some form of informal social support enjoy some health benefits during the most stressful times of their condition. However, an overly meddling social support could exacerbate the stress associated with diabetes management among PLWD [9]. PLWDs may be overwhelmed by the pieces of advice and or interferences by their support groups. Hence, while it may be well-intentioned, informal social support can sometimes be perceived as directive by the recipient, which may have inadvertent consequences on diabetes management. Evidence reveals that social support is an important moderator of any intervention [31] but has been less commonly employed in diabetes studies. Because lifestyle factors are the key in preventing the devastating complications of diabetes and lifestyle behaviors must be patient-driven, it is imperative that we actively engage individuals in the management of their disease. In the resource limited setting, social support may be a promising approach toward improving self-care and improving overall health and wellbeing [32]. Thus, there is a potential value in designing interventions to enhance social support among PLWD to reduce depressive symptomatology, improve diet and medication adherence, and increase overall wellbeing.

### Diet related NCDs landscape in Ghana

Ghana is a lower-middle income country experiencing an epidemiological transition in its disease profile [33, 34]. In 2017, 518,400 Ghanaians were estimated to be living with diabetes and a prevalence rate estimated to be 5%, with about 9,778 diabetes related deaths recorded (among ages 20–79 years) in 2017 [1]. According to the records unit of the Tamale Central Hospital, the total number of diabetes patients who visited the hospital for the years 2016 and 2017 were 1,065 and 2,023 respectively [35]. Similar trends have been observed in the Upper West and Upper East regions even though prevalence data is scarce in these regions. A combination of economic policies, rapid urbanization, and growth in the service sector have led to increased sedentary lifestyles and consumption of unhealthy diets. Not surprisingly, urbanization has also increased with time as an estimated 50% of people now live in major cities [36]. Since the 1980s, trade liberalization policies and globalization economic forces have also contributed to a growing processed food industry, both national (e.g. Papaye) and transnational (e.g. KFC) as well as an influx of shopping malls (e.g. A & C, Accra, Kumasi & West Hills malls) with cheap, convenient, energy-dense and high-protein foods [34]. These socioeconomic changes are creating a conducive climate for NCDs (e.g. cardiovascular diseases, diabetes, cancer) to thrive as people are increasingly exposed to the risk factors that increase disease susceptibility. Recent estimates indicate that NCDs are responsible for about 42% of deaths [37] and across the country, out-patient records show high reported incidence of hypertension, diabetes, and sickle cell in health facilities between 2011 and 2014 [38]. As lifestyle and diets increasingly mirror those in western societies, the burden of chronic diseases is expected to rise, which is prompting interest from both public health officials and non-health actors (e.g., traditional leaders, faith-based organizations, media institutions). However, with few exceptions [10, 39–41], little attention is paid to the changing social and cultural dynamics, and their contributions to the rise of NCDs and NCDs management in Ghana.

### Methods and data

Data were collected through a cross-sectional survey conducted from December 2022 to February 2023. Participants in this study were adults living with diabetes (persons above 18 years) in the Northern, Upper East and Upper West regions. The in-person administered survey tool contained questions pertaining to demographics, diabetes education, diabetes care, peer-support, diet adherence, exercise and food access barriers, and attitudes toward diabetes. The exercise and food access barriers related questions were modified to reflect locally available sources of exercise and food. People living with diabetes, aged 18 and above partaking in hospital-based diabetes management programs, were purposely selected from the three regions. Based on a 5% error margin, and a 95% confidence range, we arrived at a sample size of 400 with enough statistical power. Our in-person survey targeted a sample of 600 PLWD, however, 522 PLWD participated in the study, representing 87% response rate. Our exclusion criteria were PLWD who are less than 18 years and those who refused to participate. The survey was administered by 15 trained research assistants (RAs) familiar with the local languages (English, Dagaare, Dagbani or Frafra). These RAs were university graduate students and were familiar with the local context. The RAs also received rigorous training that focused on the research objectives, what each question in the questionnaire sought to measure and general ethical considerations in the data collection process. All participants completed an in-person verbal consent process prior to beginning the survey.

### Ethics and data access

This research was approved by Queen’s University Ethics Review Board (GREB Ref #: GSKHS-412–22). In Ghana, ethical approval was granted by the Ethics Committee of the Research and Development Division of Ghana Health Service (GHS-ERC 030/07/22). All methods were performed in accordance with relevant guidelines and regulations and in accordance with the Declaration of Helsinki. The study purpose was explained to the participants and verbal consent obtained before data collection. Additionally, legally Authorized Representatives of illiterate participants (e.g., family members) provided informed consent for the study. An explicit statement indicates that continuing to the questions implied informed consent to participate in the study. Participants were also told they could opt out completely at any time and that they could choose not to answer specific questions without any penalty. The researchers were external to the operations of the diabetes programs and had no control over participants’ access to care and other resources.

## Measures

### Outcome variables

Table 1 presents the outcomes and explanatory variables used in this study. Understanding diabetes management was measured using 10 questions on a 5-point Likert scale that asked participants to rate their understanding of: 1) diet control, 2) blood sugar control, 3) weight management, 4) exercise, 5) use of insulin, 6) footcare, 7) eye care, 8) diabetes complications, 9) medication and 10) alcohol use. An index was then created based on participants’ scores on the 10 questions and dichotomized as ‘0’ deemed as inadequate understanding of diabetes (for scores 0—20), and ‘1’ as adequate understanding of diabetes (scores 21—40). The second outcome variable, ability to manage diabetes, was measured using ‘yes’ or ‘no’ questions that asked participants to rate their ability to: 1) ‘keep my blood sugar in good control’, 2) ‘keep my weight under control’, 3) ‘do the things I need to do for my diabetes and 4) ‘handle my feeling’. A diabetes management index (0—4) was created and participants who scored 4 were deemed as being able to effectively manage their diabetes, coded ‘1’ and scores of 0—3 were deemed as unable to effectively manage their diabetes, coded ‘0’.

**Table 1:** Descriptive statistics

Variables	Frequency (%)
Understanding diabetes management	
Poor	205(39.65)
Good	312(60.35)
Diabetes management	
No	346(66.92)
Yes	171(33.08)
Perceived Social capital	
Weak	215(41.59)
Strong	302(58.41)
Age of respondent	
20–45	110(21.28)
46–60	213(41.20)
61–94	194(37.52)
Gender	
Male	192(37.14)
Female	325(62.86)
Ethic group	
Dagaaba	104(20.12)
Dagomba	146(28.24)
Frafra	46(8.90)
Gonja	29(5.61)
Gurisi	25(4.84)
Waala	33(6.38)
Kasina	16(3.09)
Others	118(22.82)
Marital status	
Married	278(53.77)
Not married	239(46.23)
Educational level	
None	261(50.48)
Primary/secondary	143(27.66)
Higher education	113(21.86)
Religious affiliation	
Christian	143(27.66)
Muslim	365(70.60)
Traditionalist	9(1.74)
Employment	
Employed	135(26.11)
Self-employed	185(35.78)
Employed	78(15.09)
Domestic work	119(23.02)
Total monthly income	
Under 300	236(45.65)
301–1000	134(25.92)
1001–2000	111(21.47)
Above 2000	36(6.96)
Household wealth	
Poorer	152(29.40)
Poor	170(32.88)
Rich	195(37.72)
Food access barriers	
Low	74(14.31)
Medium	353(68.28)
High	90(17.41)
Exercise barriers	
Low	200(38.68)
Medium	168(32.50)
High	149(28.82)
Diet adherence	
No	246(47.58)
Yes	271(52.42)
Commodities	
None	246(47.58)
One health condition	234(45.26)
2 or more health conditions	37(7.16)
Observations	517

### Independent variables

We employed a modified measure of perceived social support using the personal resource questionnaire. PLWD were asked to indicate their level of agreement along a 7-point Likert scale from 1 ‘strongly disagree’ to 7 ‘strongly agree’ with their perceived support from close relationships, family, relatives, and groups. An index of perceived social support was then created based on respondent’s scores on all the 15 items. Scores between 0–42 were deemed as weak, coded ‘0’ while scores above 43 were deemed as strong and coded ‘1’. Socio-demographics factors controlled for include: age (0 = 20–45; 1 = 46–60; 2 = 61 +), gender (0 = male; 1 = female), ethnic group (0 = Dagaaba; 1 = Dagomba; 2 = Frafra; 3 = Gonja; 4 = Gurisi; 5 = Waala 6 = Kasina 7 = Others), marital status (0 = married; 1 = not married),, religious affiliation (0 = Christian; 1 = Muslim; 2 = Traditionalist), employment status (0 = unemployed; 1 = self-employed, 3 = employed, 4 = Domestic work), and level of education (0 = none; 1 = primary/secondary; 2 = higher education). Further, a wealth index was calculated as a function of 22 self-reported assets, including number of houses, animal ownership (e.g., cattle, goats, chicken etc.), motorized vehicles and bicycles, and other household amenities (e.g., fridge, television, computer, cell phone etc.). Each asset was standardized before principal component analysis was used to calculate a wealth score for each household [42]. Diabetes management and health related variables such as diet adherence (0 = no; 1 = yes), food access barrier (0 = low; 1 = medium; 2 = high), exercise barriers (0 = low; 1 = medium; 2 = high), commodities (0 = none; 1 = one health condition; 2 = 2 or more health conditions) and general health were also controlled for.

### Data analysis

Based on the distribution of our dependent variables, generalized linear latent and mixed models (gllamm) with binomial and a logit(log) link function were used to analyze participants' understanding of diabetes management. Gllamm was employed to correct for any bias in the standard errors and parameter estimates due to the hierarchical nature of the data which violates the assumption of independence of respondents in standard logistic regression (see [43, 44]). In our analytical strategy, we first provided the means and proportion of each variable. Second, we assessed the multivariate relationships between the potential cofounders and our outcome variables (participants' understanding of diabetes management and their ability to manage their diabetes). Selection of independent variables for our analysis was influenced by theoretical relevance, data availability, statistical significance, and prior research on diabetes management in underserved communities. Model 1 of our analysis controlled only social capital and demographic variables while model 2 added diabetes management related variables.

## Results

### Characteristics of participants

As shown in Table 1, 60% of participants reported their understanding of diabetes management to be good whereas only 33% indicated they can effectively manage their diabetes. About 58% of participants reported high levels of perceived social capital. Majority of the participants were female. About 28% and 20% reported Dagomba and Dagaaba as their ethnic group respectively. Approximately 54% and 51% of participants reported being currently married and no education, respectively. About 71% of participants identified as Muslims, with most participants being self-employed. Regarding food and exercise barriers, 68% of participants reported medium access barriers, while about 60% reported either medium (32.50%) or high (28.82%) exercise barriers in their communities. About 52% of participants reported adhering to their dietary recommendations and about 52% indicated they had an additional health condition in addition to diabetes.

#### Determinants of understanding diabetes management

The multivariate results examining PLWD's understanding of diabetes management are shown on Table 2. In model 1, the results indicate that strong perceived social support (OR = 3.52, *p* ≤ 0.01) was associated with good understanding of diabetes management compared to PLWD with weak perceived social support. Other factors associated with diabetes management in model 1 included ethnicity, total monthly income, and household wealth. For instance, people who identify as belonging to Dagomba (OR = 0.19, *p* ≤ 0.01), Frafra (OR = 0.07, *p* ≤ 0.01), Gonja (OR = 0.07, *p* ≤ 0.01), Gurisi (OR = 0.21, *p* ≤ 0.05), Kasina (OR = 0.02, *p* ≤ 0.01) and other (OR = 0.08, *p* ≤ 0.01) ethnic groups had lower odds of reporting good understanding of diabetes management compared to Dagaaba. In model II, the association between perceived understanding diabetes management and social support (OR = 2.27, *p* ≤ 0.05) was attenuated but remained significant. In addition to age, ethnic group, total income, household wealth, exercise, and food barriers, we found that PLWD with one (OR = 3.79, *p* ≤ 0.01) or two or more (OR = 8.86, *p* ≤ 0.01) comorbid health conditions had greater odds of reporting good understanding of diabetes management compared to those with only diabetes.

**Table 2:** Multivariate determinants of PLWDs understanding diabetes management

	Model 1	Model 2
Independent variables	Diabetes Management	Diabetes Management
Social capital (ref: weak)	OR (95%CI)	OR (95%CI)
Strong	3.52(1.89—6.53)***	2.27(1.19—4.33)**
Age of respondent (ref:20–45)		
46–60	0.50(0.24—1.06)*	0.63(0.29—1.32)
61–94	0.60(0.25—1.41)	0.33(0.13—0.79)**
Gender (ref: Male)		
Female	0.74(0.40—1.37)	0.58(0.31—1.08)*
Ethic group (ref: Dagaaba)		
Dagomba	0.19(0.06—0.54)***	0.49(0.17—1.39)
Frafra	0.07(0.02—0.31)***	0.21(0.05—0.81)**
Gonja	0.07(0.01—0.33)***	0.19(0.04—0.84)**
Gurisi	0.21(0.05—0.92)**	0.78(0.17—3.55)
Waala	2.40(0.54—10.68)	2.13(0.48—9.44)
Kasina	0.02(0.003—0.14)***	0.04(0.01—0.27)***
Others	0.08(0.03—0.27)***	0.31(0.09—1.01)*
Marital status (ref: married)		
Not married	1.22(0.67—2.21)	1.62(0.88—2.98)
Educational level (ref: None)		
Primary/secondary	2.00(0.99—4.03)*	1.93(0.95—3.91)*
Higher education	2.32(0.75—7.11)	2.74(0.85—8.82)*
Religious affiliation (ref: Christian)		
Muslim	0.74(0.34—1.60)	1.15(0.52—2.54)
Traditionalist	3.02(0.41—22.13)	3.01(0.35—25.53)
Employment (ref: unemployed)		
Self-employed	0.79(0.32—1.94)	0.65(0.26—1.61)
Employed	0.65(0.19—2.14)	0.61(0.17—2.12)
Domestic work	2.02(0.830—4.959)	1.48(0.60—3.62)
Total monthly income (ref: under 300)		
301–1000	3.01(1.23—7.34)**	2.98(1.24—7.18)**
1001–2000	2.24(0.82—6.11)	2.17(0.81—5.87)
Above 2000	41.32(3.41—499.7)***	43.98(3.60—536.5)***
Household wealth (ref: poorer)		
Poor	3.35(1.64—6.83)***	2.13(1.03—4.37)**
Rich	5.10(2.28—11.41)***	3.36(1.47—7.68)***
Food access barriers (ref: low)		
Medium		0.83(0.38—1.77)
High		4.09(1.32—12.59)**
Exercise barriers (ref: low)		
Medium		0.86(0.42—1.79)
High		0.17(0.06—0.45)***
Diet adherence (ref: no)		
Yes		1.62(0.87—3.01)
Commodities (ref: no)		
One health condition		3.79(1.99—7.23)***
2 or more health condition		8.86(2.78—28.15)***
Disability (ref: no)		
Yes		1.13(0.54—2.36)
Constant	1.44(0.27—7.63)	0.53(0.07—3.59)
	3.11(1.54—6.26)***	2.14(1.02—4.47)**
Observations	517	517

*OR* Odds ratio, *Ref* Reference Categories; *p ≤ .10, **p ≤ .05, ***p ≤ .01; *CI* confidence intervals

#### Determinants of PLWD's ability to manage diabetes

The multivariate results examining PLWD's ability to manage diabetes are shown on Table 3. In model 1, the results indicate PLWD with strong perceived social support (OR = 2.34, *p* ≤ 0.01) had greater odds of being able to manage their diabetes compared to PLWDs with weak perceived social support. Other factors associated with diabetes management in model 1 included ethnicity, and household wealth. For instance, people who identify as belonging to Dagomba (OR = 0.06, *p* ≤ 0.01), Frafra (OR = 0.11, *p* ≤ 0.01), Gonja (OR = 0.11, *p* ≤ 0.01), Gurisi (OR = 0.04, *p* ≤ 0.05), Kasina (OR = 0.11, *p* ≤ 0.01) and other (OR = 0.09, *p* ≤ 0.01) ethnic groups had lower odds of being able to manage their diabetes compared to Dagaaba. In model II, the association between perceived ability to manage diabetes and social support (OR = 2.14, *p* ≤ 0.05) was attenuated but remained significant. In addition, PLWD who reported medium (OR = 0.28, *p* ≤ 0.01) and high (OR = 0.09, *p* ≤ 0.01) physical activity barriers had lower odds of being able to manage their diabetes. In addition, PLWD who reported adherence to dietary recommendations were more likely to report being able to manage their diabetes. Also, PLWD with total monthly income of 301–1,000 had greater odds of being able to manage their diabetes compared to PLWD with incomes less than or equal to 300. Ethnic group was associated with ability to manage diabetes.

**Table 3:** Multivariate determinants of PLWDs ability to manage diabetes

	Model 1	Model 2
Variables	Diabetes Management	Diabetes Management
Social capital (ref: weak)	OR (95%CI)	OR (95%CI)
Strong	2.34(1.37—3.96)***	2.14(1.16—3.97)**
Age of respondent (ref:20–45)		
46–60	1.48(0.78—2.81)	1.56(0.77—3.14)
61–94	1.20(0.56—2.53)	1.24(0.54—2.82)
Gender (ref: Male)		
Female	1.01(0.581—1.746)	0.88(0.47—1.63)
Ethic group (ref: Dagaaba)		
Dagomba	0.06(0.03—0.14)***	0.14(0.05—0.32)***
Frafra	0.11(0.04—0.29)***	0.16(0.05—0.50)***
Gonja	0.11(0.03—0.33)***	0.37(0.09—1.37)
Gurisi	0.04(0.01—0.18)***	0.13(0.02—0.73)**
Waala	0.68(0.26—1.77)	0.72(0.25—2.04)
Kasina	0.11(0.02—0.44)***	0.25(0.05—1.23)*
Others	0.09(0.04—0.19)***	0.22(0.08—0.56)***
Marital status (ref: married)		
Not married	0.72(0.42—1.23)	0.86(0.47—1.56)
Educational level (ref: None)		
Primary/secondary	1.81(0.99—3.30)*	1.40(0.71—2.74)
Higher education	1.69(0.64—4.49)	1.96(0.67—5.71)
Religious affiliation (ref: Christian)		
Muslim	1.33(0.69—2.53)	1.47(0.73—2.97)
Traditionalist	2.61(0.52—13.11)	1.74(0.27—11.00)
Employment (ref: unemployed)		
Self-employed	0.92(0.40—2.06)	1.07(0.44—2.60)
Employed	0.71(0.26—1.87)	0.62(0.22—1.77)
Domestic work	1.49(0.64—3.50)	1.82(0.71—4.64)
Total monthly income (ref: under 300)		
301–1000	2.06(0.94—4.50)*	2.36(1.02—5.46)**
1001–2000	1.81(0.74—4.42)	1.87(0.71—4.93)
Above 2000	2.46(0.68—8.86)	1.90(0.47—7.730
Household wealth (ref: poorer)		
Poor	1.23(0.602—2.540)	0.81(0.35—1.87)
Rich	4.09(1.93—8.65)***	1.77(0.73—4.26)
Food access barriers (ref: low)		
Medium		1.58(0.769—3.258)
High		1.72(0.70—4.21)
Exercise barriers (ref: low)		
Medium		0.28(0.15—0.52)***
High		0.09(0.03—0.27)***
Diet adherence (ref: no)		
Yes		2.85(1.54—5.31)***
Commodities (ref: no)		
One health condition		0.81(0.46—1.42)
2 or more health condition		0.34(0.09—1.29)
Disability (ref: no)		
Yes		0.79(0.36—1.73)
Constant	0.29(0.06—1.25)*	0.24(0.03—1.60)
	1.00(0.42—2.34)	1.00(0.55—1.78)
Observations	517	517

*OR* Odds ratio, *Ref* Reference Categories; *p ≤ .10, **p ≤ .05, ***p ≤ .01; *CI* confidence intervals

## Discussion

This study examined the complex challenges associated with diabetes management in an underserved setting in a lower- middle income country with emphasis on the role of social support and built environment factors. Our findings showed that even though the majority of people living with diabetes report adequate diabetes management knowledge, only a few were able to manage the chronic condition. Diabetes management is an enduring process and requires constant care to achieve and maintain optimal glycemic control. However, continuing vigilance is stressful for PLWD. The findings from this research lend support to the role of social support in facilitating the adoption of a healthful lifestyle including diabetes management [23, 29]. For instance, a study conducted in South Africa by Werfalli and colleagues [29] found that family support has a positive association with self-management practice among older persons with Type 2 diabetes. The respondents, who were attending primary care clinics in Cape Town, reported that family support (particularly from children and spouses) was integral in the daily self-management of their condition. The support givers supported the respondents with reminders to stick to meal/physical activity plans, to test blood sugars and to handle the feelings of having diabetes. Similarly, in assessing the impact of social support and relationships with the health care system among low-income populations in Buffalo, New York, Vest et al. [23] noted that respondents who reported having some social support networks were likely to feel comfortable and more confident in managing their diabetes. Comfortability and confidence were related to motivation given and the physical support in acquiring some resources for the management of diabetes. Vest and colleagues [23] further asserted that, usually, concern for the health of people in one’s social network brings to bear the intrinsic motivation of PLWD to fortify diabetes self-management habits. Informational social support may provide knowledge and assist PLWD in maintaining diet routines, exercising consistently; and emotional support to PLWD. Social support helps patients with chronic conditions in significant ways to improve self-care skills, knowledge of their diseases, and symptoms of distress, thereby improving their overall wellbeing [45]. Further, evidence also reveals that social support is an important moderator of any diabetes intervention. The social support often received from family members, friends, and members from one’s neighbourhood or how such support is structured can impact diabetes management [46]. Friends, family members and members of one’s community are key players in providing the daily tasks (e.g., support with physical activities and preparing meals) to meet the needs of their persons living with diabetes (PLWD) toward their wellbeing [23]. In this context, social support is a promising approach toward improving self-care and improving overall health and wellbeing. Thus, there is a potential value in designing interventions to enhance social support among PLWD to reduce diabetes-related depression, co-manage diabetes-associated NCDs and increase overall wellbeing. Enhancing social support can offer consistent and substantial care and psychological support to PLWD. Given the increasing burden of diabetes in LMICs, and the lack of targeted prevention strategies coupled with inadequate care for PLWD, there is an urgent need to identify context relevant interventions with the potential to improve diabetes management outcomes among patients with diabetes and preventing diabetes among individuals at high risk.

Another key finding of this study is the important role of the built environment including access to physical activity, nutritious and healthy diets toward diabetes management [39, 47–49]. Our findings indicate that PLWD who lived in environments with high physical activity access barriers were less likely to report good understanding of diabetes management. The food environment within which PLWD live also places constraints on PLWD's perceived control of their diabetes situation. Even though PLWD may want to undertake the recommended daily physical activity and nutritional requirements, the built environment places limitations on PLWD's perceived control of their diabetes and affects the extent to which PLWDs can undertake these activities. Even though it is recommended that people with diabetes obtain at least 150 min of moderate to vigorous aerobic exercise per week [50, 51], the built environment may act as an external control factor limiting PLWD's ability to exercise. In Ghana, as in many other African, countries, fruit, and vegetable consumption is low, with a significant proportion of adults (86%), falling short of the WHO's recommended 400gm/day or 5 servings/day. Even though Ghana’s national strategy on NCD prevention aims to increase: (a) awareness about healthy diets through health promotion; and (b) the availability of healthier foods, major challenges exist including inefficient program management, low funding, lack of political interest, and low community awareness [10, 11]. Our work supports previous research that explored the contributions of the changing household and community environments to the rise of NCDs and impede NCDs management [39–41]. Dake et al. [39], for example demonstrate that the urban food environment in Accra is characterized by an abundance of out-of-home cooked foods, convenience stores, and limited fruits and vegetables options, which is, as the authors highlight, contributing to high body mass index among urban poor residents. In other urban centers such as Kumasi, whereas people perceive that fast foods are unhealthy, expensive, or too foreign, others also indicate they are convenient, delicious, save time and good for fun and change [52]. While collectively, these studies are important in painting a picture of ongoing changes in diets, our research will also contribute to enhance knowledge and understanding around the built environment and diabetes management especially among PLWD in marginalized and rural communities.

Our findings revealed that the majority (52%) of PLWD reported living with other health conditions and, compared to PLWD with no other health condition, those with another health condition were more likely to perceive their diabetes management as good. PLWD with comorbidities are therefore likely to perceive their condition as serious and to adopt attitudes to effectively manage diabetes. On the other hand, PLWD with diabetes-related comorbidities and other conditions they consider to be unrelated to diabetes may need additional support in making decisions about care priorities and self-management activities. Thus, to ensure a comprehensive approach to diabetes management, the presence of multimorbidity should be considered in the context of clinical decision making. However, to better meet the needs of PLWD and comorbidities, more research is needed to determine the risks and benefits of an integrated and health system-based approaches to NCDs management. Focus needs to be given to structurally exposed communities while ensuring that interventions are more sustainable, equable, and balanced [53, 54].

There are potential limitations associated with this study. Our study suffers potential Berkson bias because it was conducted among PLWD participating in a hospital-based diabetes program and our sample was not drawn from the general population. Since our study was conducted in a hospital setting, it is also possible that PLWDs with limited social capital and social support were more likely to participate in these hospital programs. Also, our study relied on self-reported experiences of diabetes management which is subject to several biases including recall bias. Also, even though we were thorough in our data collection and analysis process and controlled for theoretically relevant covariates, it is possible that the relationships between our independent and outcome variables reflected the unmeasured influence of other omitted variables. Despite these limitations, the findings have implications for promoting the overall well-being of people living with diabetes in resource-poor settings including Ghana and other sub-Saharan countries and contribute toward achieving the Sustainable Development Goals 3 (SDGs).

## Conclusion

The overarching goal of the study was to assess diabetes management and supportive care needs of PLWD in underserved communities. Overall, the study demonstrates the important role of social support networks, the built environment and comorbidities in diabetes management and points to the need for an integrated, community-led and health-systems approach to diabetes management. To further understand how social support may influence diabetes management, qualitative study may be needed to examine how social support influences different factors to enhance or impede the management of diabetes. The research provided evidence to inform strategies to support diabetes management and prevention in Ghana. For example, by unpacking the social and environmental factors in the production of inequality in diabetes management, decision makers and practitioners will be able to better design interventions that remove or minimize barriers to diabetes management. Further, while the results are context-specific to Ghana, opportunities exist for policy makers to draw on the findings to shape the design of future studies to capture patterns and relationships of various diabetes management social determinants in LMICs. In resource poor settings such as ours, creating supportive communities and networks of support for PLWD may contribute towards enhancing self-care and improving overall health and wellbeing of PLWD [32]. Thus, there is a potential value in designing interventions to enhance social support among PLWD to reduce depressive symptomatology, improve diet and medication adherence, and increase overall wellbeing. Overall, this research will also contribute to wider discussions on changing local food environments and the role they play in the rise of diet-related diseases as well as how to modify attitudes and behaviors around diabetes and NCDs management as many LMICs experience rapid epidemiological and nutrition transition.

## Data Availability

The datasets generated and/or analysed during the current study are not publicly available as ethics approval allowed access only for the named researchers (the authors), however ethics amendments to make data available will be considered upon reasonable request made to the corresponding author.

## Abbreviations

LMICs: Low- and Middle-Income Countries
NCDs: Non-communicable diseases
PLWD: Persons living with Diabetes Miletus
SSA: Sub-Saharan Africa
